# Clinicopathological Features and Outcomes of Endoscopic Submucosal Dissection for Early Gastric Lymphoepithelioma-like Carcinoma

**DOI:** 10.3390/cancers17183050

**Published:** 2025-09-18

**Authors:** Young Eun Oh, Tae-Se Kim, Yo Han Jeon, Soomin Ahn, Kyoung-Mee Kim, Yang Won Min, Hyuk Lee, Byung-Hoon Min, Jun Haeng Lee, Poong-Lyul Rhee, Jae J. Kim

**Affiliations:** 1 Department of Medicine, Samsung Medical Center, School of Medicine, Sungkyunkwan University, 81 Irwon-ro, Gangnam-gu, Seoul 06351, Republic of Korea; dhduddms803@naver.com (Y.E.O.);; 2 Department of Pathology and Translational Genomics, Samsung Medical Center, School of Medicine, Sungkyunkwan University, 81 Irwon-ro, Gangnam-gu, Seoul 06351, Republic of Korea

**Keywords:** stomach neoplasms, endoscopic resection, lymphoepithelioma-like carcinoma

## Abstract

Background/Objectives: The curability of endoscopic submucosal dissection (ESD) for early gastric lymphoepithelioma-like carcinoma (LELC) remains unclear, as this rare histological subtype is not well represented in current guidelines. We aimed to evaluate the clinicopathological features and outcomes of early gastric LELC following ESD. Methods: We retrospectively compared 51 patients with early gastric LELC and 8243 patients with well- or moderately differentiated (WD or MD) tubular adenocarcinoma who underwent ESD. Results: LELC was more frequently located in the proximal stomach than WD/MD adenocarcinoma (52.9% vs. 10.3%). The deep submucosal invasion rate was significantly higher in LELC (77.3% vs. 9.5%), whereas the lymphatic invasion rate was comparable between the two groups (5.7% vs. 9.2%). No LELC cases were diagnosed preoperatively using forceps biopsy. Despite the comparable en bloc with R0 and complete resection rates, the curative resection rate of LELC was significantly lower than that of WD/MD adenocarcinoma, primarily due to deep submucosal invasion. Post-ESD bleeding was more frequent in LELC (11.3% vs. 2.7%). During a mean follow-up of 38.1 months, no extra-gastric recurrence was observed in patients who did not undergo additional surgery, and no lymph node metastasis was detected among the 23 patients who underwent gastrectomy. No gastric cancer-specific deaths occurred in the study population during follow-up. Conclusions: Considering these favorable long-term outcomes despite a low curative resection rate, early gastric LELC fulfilling the conventional curative criteria of current guidelines can be regarded as having been curatively treated by ESD, particularly in patients with high surgical morbidity.

## Simple Summary

Gastric lymphoepithelioma-like carcinoma (LELC) is a rare variant of gastric cancer, known for having unique histopathological features and better prognosis than other subtypes of gastric cancer. However, due to its rarity, current guidelines for endoscopic submucosal dissection (ESD) do not provide specific curability criteria or treatment recommendations for early gastric LELC. Furthermore, studies on both short- and long-term outcomes of ESD for early gastric LELC remain limited. Therefore, we investigated the clinicopathological features and outcomes of early gastric LELC following ESD. Despite frequent deep submucosal invasion leading to non-curative resections, early gastric LELC achieves favorable long-term outcomes after ESD. Given these findings, we suggest that ESD could be considered a curative option in carefully selected patients, especially those with high operative risk, to avoid invasive overtreatment.

## 1. Introduction

Gastric lymphoepithelioma-like carcinoma (LELC), also known as gastric carcinoma with lymphoid stroma or medullary carcinoma, is a rare histologic subtype of gastric cancer, which is characterized by irregular sheets, poorly defined clusters or tubules, trabeculae, or syncytial cells with dense lymphocytic infiltration and intraepithelial lymphocytes [1]. LELC accounts for only 1–4% of all gastric cancer cases [2,3,4] and is often associated with Epstein–Barr virus (EBV) infection [5,6,7,8]. Despite increasing recognition of early gastric LELC, current endoscopic submucosal dissection (ESD) guidelines in Japan and Korea do not provide specific curability criteria or treatment recommendations for this rare subtype [9,10]. Due to the low diagnostic yield of pre-ESD forceps biopsy [11,12], LELC is typically diagnosed only after ESD, leaving clinicians without established curability criteria to guide decisions regarding the need for further management.

In our previous study of patients with early gastric LELC who underwent gastrectomy, deep submucosal invasion was more common in early gastric LELC than in well- or moderately differentiated (WD or MD) early gastric cancers (EGCs). Despite this aggressive feature, the risk of lymph node metastasis (LNM) in early gastric LELC remained negligible when the tumor was confined to the mucosa or shallow submucosa layer [13]. Furthermore, studies on advanced gastric LELC have consistently reported lower LNM rates and better surgical outcomes compared to other histologic subtypes [14,15,16,17]. These findings suggest that ESD may serve as a potential curative option for early gastric LELC. However, evidence on both the short- and long-term outcomes of ESD for early gastric LELC remains limited. To date, the two largest published series on ESD for early gastric LELC included only 18 and 40 cases, respectively [12,18]. Therefore, further data are needed to clarify the clinical outcomes of early gastric LELC after ESD and to determine whether the current curability criteria for ESD can be safely applied to this rare subtype.

This study aimed to compare the clinicopathological features of early gastric LELC with those of WD or MD EGCs treated with ESD. We also investigated the treatment outcomes and long-term clinical course of patients with early gastric LELC after ESD.

## 2. Materials and Methods

### 2.1. Patients

**Figure 1:**
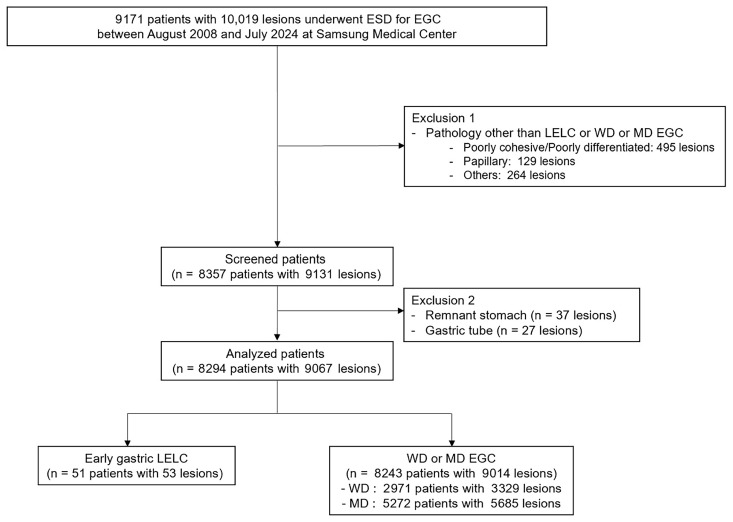
Flowchart of the study population. EGC, early gastric cancer; ESD, endoscopic submucosal dissection; LELC, lymphoepithelioma-like carcinoma; WD, well-differentiated; MD, moderately differentiated.

From August 2008 to July 2024, a total of 9171 patients with 10,019 EGCs underwent ESD at Samsung Medical Center. Among them, 814 patients with 888 lesions were excluded due to histologic diagnoses other than early gastric LELC, WD or MD tubular adenocarcinoma. Additionally, 63 patients with 64 EGCs arising from the remnant stomach or gastric tube were excluded. Finally, 51 patients with 53 lesions of early gastric LELC and 8243 patients with 9014 lesions of WD or MD EGC were included in the analysis (Figure 1). Demographic and clinicopathological data were extracted from medical records using the intranet resources of Samsung Medical Center. The Institutional Review Board (IRB) of Samsung Medical Center approved this study (approval number: 2025-05-154, approval date: 9 June 2025). This study was conducted in accordance with the guidelines of the Declaration of Helsinki. The requirement for informed consent was waived by the IRB.

### 2.2. ESD Procedures and Histopathological Evaluation

The ESD procedures conducted at our institution have been previously detailed [19,20]. Resected specimens were carefully stretched, secured to a polystyrene plate, and fully immersed in 10% neutral buffered formalin for at least 12 h to ensure adequate fixation. After fixation, the specimens were serially sectioned at 2 mm intervals, parallel to the closest resection margin to assess both lateral and vertical margins. The depth of invasion, lymphovascular involvement and histologic differentiation were then evaluated [21].

LELC was characterized by distinct histopathological features: (i) a well-demarcated tumor margin; (ii) dense lymphocytic infiltration, with tumor-infiltrating lymphocytes consistently outnumbering neoplastic cells; (iii) indistinct cytoplasmic borders and a syncytial growth pattern with poorly formed glandular structures; and (iv) absence of a desmoplastic stromal reaction [15].

### 2.3. EBV-Encoded RNA in Situ Hybridization

In situ hybridization is the most suitable and widely used method for detecting EBV in formalin-fixed, paraffin-embedded specimens. Tissue sections 3 µm thick were prepared from each block and mounted on Superfrost Plus slides (Thermo Scientific, Waltham, MA, USA). In situ hybridization was performed using a fully automated BOND-MAX system with an EBV-encoded RNA probe (Leica, Newcastle, UK), following the manufacturer’s guidelines. Sections were classified as EBV-positive only when all tumor cell nuclei exhibited a strong signal.

### 2.4. Definitions

En bloc resection was defined as the excision of the tumor in a single specimen without any endoscopically detectable residual tumor. R0 resection was defined as complete tumor excision with histologically confirmed tumor-free lateral and vertical margins. Complete resection was defined as en bloc resection with negative lateral and vertical resection margins, in the absence of lymphovascular and perineural invasion. Curative resection was defined as complete resection fulfilling one of the following criteria for WD or MD EGCs: (i) mucosal tumor ≤2 cm without ulceration; (ii) mucosal tumor >2 cm without ulceration; (iii) mucosal tumor ≤3 cm with ulceration; or (iv) submucosal tumor ≤3 cm with invasion <500 μm from the muscularis mucosae (SM1 cancer). Currently, no established ESD curability criteria exist for gastric LELC. In addition, gastric LELC cannot be classified pathologically as a differentiated or undifferentiated-type tumor. Therefore, curative resection of early gastric LELC was assessed using both the criteria for differentiated- and undifferentiated-type EGC. Curative resection of an undifferentiated-type tumor was defined as complete resection of a mucosal tumor ≤2 cm in size without ulceration.

Current Helicobacter pylori infection was considered positive when at least one of the following tests yielded positive results: the 13C urea breath test, rapid urease test, or histological examination with Giemsa staining.

Post-ESD bleeding was defined when one of the following conditions was met within 4 weeks after ESD: (i) clinical signs of gastrointestinal bleeding, including hematemesis, melena, or hematochezia, requiring endoscopic, radiologic, or surgical intervention; or (ii) a decrease in hemoglobin level of >2 g/dL after ESD. Perforation was diagnosed based on either of the following criteria: (i) frank perforation, defined as endoscopic identification of mesenteric fat or direct exposure of the peritoneal cavity during ESD; or (ii) microperforation, defined as the detection of free intraperitoneal air on radiography or computed tomography (CT) in the absence of an endoscopically visible gastric wall defect.

Long-term outcomes following ESD, including both local and extra-gastric recurrence, were evaluated in all the patients who underwent ESD for early gastric LELCs. Residual lesions were defined as tumors identified at the primary resection site during the first or second follow-up EGD within 12 months after resection. Local recurrence was defined as the detection of cancer at the primary resection site after R0 resection. Gastric tumors detected at sites other than the primary resection site within 12 months after ESD were defined as synchronous lesions, whereas those detected at least 12 months after ESD were classified as metachronous lesions. Extra-gastric recurrence was defined as the presence of cancer in lymph nodes beyond the locoregional area and/or in distant organs, as determined by CT and confirmed by pathological evaluation.

### 2.5. Follow-Up After ESD

EGD with biopsy was performed 2 months after ESD to evaluate healing of the artificial ulcer and to exclude residual tumor. Patients underwent surveillance with EGD and biopsy, along with abdominal CT, at 6-month intervals for the first 3 years and annually thereafter during years 4 and 5 post-ESD. Follow-up duration was defined as the interval from the date of ESD to the date of the last surveillance endoscopy, imaging study, or clinical visit.

### 2.6. Statistical Analysis

Categorical variables were compared using the χ^2^ test and Fisher’s exact test, and continuous variables were evaluated with Student’s *t*-test and Mann–Whitney U test, as appropriate. Two-sided *p* values < 0.05 were considered statistically significant. Analyses were performed using SPSS version 29.0 (IBM Corp., Armonk, NY, USA).

## 3. Results

### 3.1. Clinicopathological Features of Early Gastric LELC Compared to WD or MD EGC

The study population included 51 patients with 53 early gastric LELCs and 8243 patients with 9014 WD or MD EGCs. Clinicopathological characteristics of early gastric LELC versus WD/MD EGC are summarized in Table 1. Early gastric LELCs were more commonly located in the proximal stomach compared with WD/MD EGCs. Notably, deep submucosal invasion (SM2 or SM3) occurred more frequently in early gastric LELC than in WD/MD EGCs (77.3% vs. 9.5%, *p* < 0.001), whereas lymphatic invasion rates were comparable between the groups (5.7% vs. 9.2%, *p* = 0.376).

**Figure 2:**
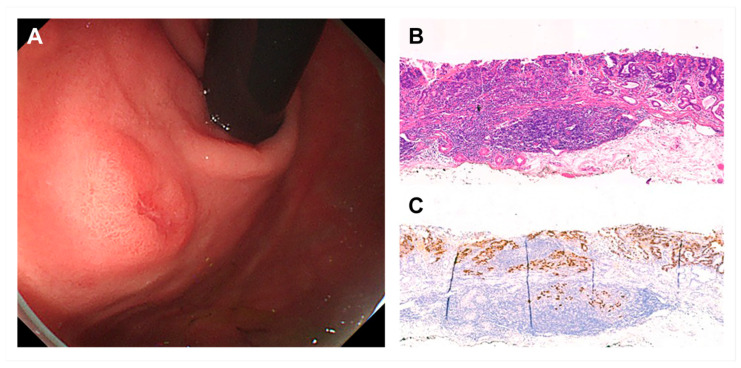
Representative macroscopic and microscopic findings of early gastric lymphoepithelioma-like carcinoma (LELC). (**A**) Typical endoscopic appearance showing a mixed-type lesion (IIa + IIc). (**B**) Histological features demonstrating poorly differentiated carcinoma with dense lymphoid stroma (×10, hematoxylin and eosin stain). (**C**) Nuclei of carcinoma cells are positive by EBV in situ hybridization.

Table 2 summarizes the detailed clinicopathological features of early gastric LELCs. The mixed macroscopic type was the most common, with IIa + IIc accounting for more than half of the lesions (Figure 2). EBV and current H. pylori infection rates were 83% and 13.2%, respectively. On initial endoscopic forceps biopsy, 96.2% of the lesions were diagnosed as moderately or poorly differentiated adenocarcinoma. None of the lesions were diagnosed as early gastric LELC prior to ESD.

### 3.2. Short-Term Outcomes of ESD for Early Gastric LELC Compared to Those for WD or MD EGC

Table 3 summarizes the short-term outcomes of ESD for early gastric LELC compared to those for WD or MD EGC. The rates of en bloc resection, R0 resection, en bloc with R0 resection, and complete resection for early gastric LELC were 94.3%, 92.5%, 88.7%, and 83.0%, respectively, with no significant differences from the rates for WD or MD EGC.

Since early gastric LELC is not clearly classified as a differentiated or undifferentiated type pathologically according to the current guidelines [1,9], the curative resection rates were analyzed using the criteria for both types. The curative resection rates for early gastric LELC were 20.8% when applying the criteria for differentiated-type EGC and 28.3% when applying those for undifferentiated-type EGC. Both rates were significantly lower than those for WD or MD EGC (80.9%). Post-ESD bleeding occurred more frequently in early gastric LELC than in WD or MD EGC (11.3% vs. 2.7%, *p* = 0.003).

### 3.3. Long-Term Outcomes of ESD for Early Gastric LELC

**Figure 3:**
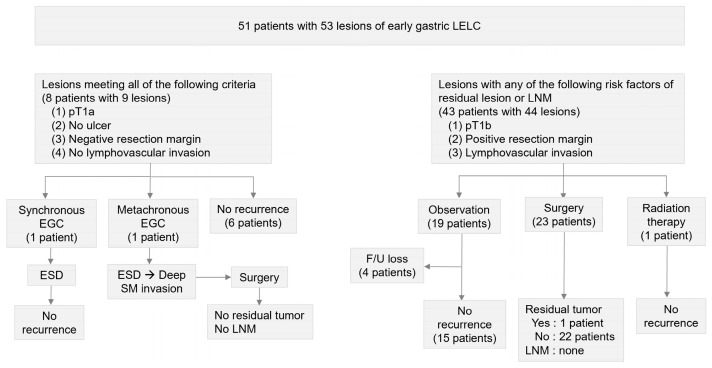
Flowchart of the follow-up outcomes of ESD for early gastric LELC. ESD, endoscopic submucosal dissection; LELC, lymphoepithelioma-like carcinoma; pT1a, mucosal cancer; pT1b, submucosal cancer; EGC, early gastric cancer; SM, submucosal; LNM, lymph node metastasis, F/U, follow-up.

Figure 3 summarizes the follow-up outcomes of ESD for early gastric LELC. Among the 51 patients who underwent ESD for 53 lesions, eight patients with nine lesions were categorized as the low-risk group, having no risk factors for residual tumor or LNM. The remaining 43 patients with 44 lesions were classified as the high-risk group, based on the presence of one or more of the following risk factors: (i) pT1b (n = 42), (ii) positive resection margin (n = 4), and (iii) lymphovascular invasion (n = 3). Over a mean follow-up period of 38.1 months (range, 2–127 months), one synchronous (tubular adenocarcinoma, MD) and one metachronous (tubular adenocarcinoma, WD) EGC were identified in the low-risk group, which were subsequently managed with curative ESD and radical gastrectomy, respectively. No local or extra-gastric recurrences were observed in this group during follow-up.

Of the 43 patients in the high-risk group, 23 patients underwent radical gastrectomy according to standard gastric cancer guidelines and one patient received adjuvant radiotherapy after ESD, while 19 patients were managed with observation. Four of these 19 were lost to follow-up, and the clinical course of the remaining 15 was analyzed (Table S1). Two patients with submucosal invasion depths of 190 μm and 250 μm were considered to have achieved curative resection and were followed with standard surveillance. Three patients with significant comorbidities and six of advanced age were observed due to excessive surgical risk, and four patients declined surgery because of concerns about postoperative complications and were managed with close observation. Pathological examination of surgical specimens revealed no LNM in the 23 gastrectomy patients; residual tumor was identified in only 1 case with deep submucosal invasion (1500 μm) and a vertical margin <50 μm. During follow-up, no lymph node or distant metastasis occurred in the high-risk group, and no gastric cancer–related deaths were observed in the entire cohort.

## 4. Discussion

Gastric LELC is a rare histologic subtype of gastric cancer that is rarely diagnosed using forceps biopsy prior to ESD. In most cases, early gastric LELC is identified only after ESD, making it a post hoc pathological diagnosis. Because current ESD guidelines do not specifically address LELC, determining curability and planning subsequent treatment can be challenging when this entity is unexpectedly encountered. In this retrospective cohort study, we aimed to clarify the clinicopathological characteristics and outcomes of early gastric LELC after ESD, with the goal of providing evidence to support the development of curability criteria for this unique histologic type. To the best of our knowledge, this study represents the largest series to date to investigate the outcomes of ESD in early gastric LELC.

Previous studies based on surgical specimens have reported that gastric LELC is typically located in the proximal stomach and often exhibits deep submucosal invasion with relatively low rates of lymphovascular invasion [13,22]. These clinicopathological characteristics were consistently observed in our cohort of early gastric LELC, reinforcing prior findings in a population treated by endoscopic resection. Few studies have characterized the gross morphology of gastric LELC, particularly early-stage disease. Previous studies on ESD outcomes for early gastric LELC have used a simplified classification system, categorizing lesions as flat, elevated, or depressed [12,18]. In contrast, our study systematically classified the endoscopic morphology using the Paris classification and newly demonstrated that early gastric LELC commonly presents as the mixed macroscopic type, with IIa + IIc being the predominant configuration.

In the present study, the rates of en bloc with R0 resection and complete resection of early gastric LELC were comparable to those of WD or MD EGCs (88.7% vs. 91.6%, *p* = 0.450; 83.0% vs. 84.6%, *p* = 0.704). These results are consistent with previous studies analyzing the ESD outcomes for differentiated-type EGC [23,24]. In fact, these results are comparable to or even better than those reported for undifferentiated-type EGCs in previous meta-analyses [25], supporting the technical feasibility of ESD in early gastric LELC. However, the curative resection rate was significantly lower in LELC, primarily due to the high frequency of deep submucosal invasion. Curative resection rates for LELC were 20.8% and 28.3% using the differentiated-type and undifferentiated-type criteria, respectively, compared to 80.9% for WD or MD EGCs. These rates were also markedly lower than the curative resection of 61.4–79.8% reported for undifferentiated-type EGCs in previous studies [25], suggesting that applying current curability criteria to early gastric LELC may result in invasive overtreatment despite the favorable clinical course observed in the present study.

Notably, post-ESD bleeding occurred more frequently in LELC than in WD or MD EGCs (11.3% vs. 2.7%). This may be attributable to the anatomic distribution of LELC lesions, with over 90% located in the upper and middle thirds of the stomach, areas known to have higher bleeding risk [24,26,27]. In addition, the higher proportion of deep submucosal dissection may have increased exposure of submucosal vessels [28,29].

Despite the lower curative resection rate, the long-term outcomes of ESD for early gastric LELC were favorable. No local or extra-gastric recurrences were observed in the low-risk group. Of the 43 patients classified as having risk factors for residual tumor or LNM, no LNM or distant metastasis was observed during the follow-up period. Additionally, no LNM was found in the surgical specimen among the 23 patients who underwent gastrectomy. Importantly, there were no gastric cancer-specific deaths in the entire study population during follow-up. Our findings are consistent with those of prior studies. Shin et al. [18] reported no recurrences during a mean follow-up of 37.2 months in 10 patients who underwent ESD for LELC. Similarly, Lim et al. [12] observed no LNM among 17 patients who underwent surgery following ESD and no extra-gastric recurrence among 23 patients managed without additional intervention after ESD for LELC. The favorable prognosis of gastric LELC may be related to its unique immune microenvironment. Previous studies have suggested that the dense lymphoid stroma and strong CD8+ T-cell infiltration in LELC contribute to enhanced anti-tumor immunity, potentially reducing the risk of nodal metastasis [15,30,31]. However, the mechanisms underlying this remain unclear and warrant further investigation.

Given its unique pathological and prognostic features, LELC should be considered an independent entity among gastric cancers. However, pre-treatment diagnosis remains challenging, even with the use of EBV PCR or adjunctive diagnostic modalities such as EUS. Therefore, in clinical practice, when LELC is suspected and ESD is technically feasible in patients with high surgical risk, ESD may serve as both a diagnostic and therapeutic option, provided that patients are thoroughly counseled regarding the potential for noncurative resection and the possible need for additional surgery.

Several previous studies have reported that mortality among patients observed after non-curative resection is most often related to comorbid conditions rather than gastric cancer itself, underscoring the importance of evaluating both gastric cancer–specific and all-cause mortality when determining the need for additional treatment [32]. In our cohort, a notable proportion of high-risk patients were observed due to advanced age or comorbidities, which were deemed to entail excessive surgical risk, and no gastric cancer–related deaths were observed during follow-up. These findings suggest that, despite the relatively low curative resection rate, early gastric LELC satisfying the conventional curative criteria may reasonably be regarded as curatively treated by ESD, particularly in patients with high surgical morbidity.

This study has some limitations. First, it was performed at a single tertiary referral center and employed a retrospective design. Second, the number of patients with early gastric LELC who underwent ESD was limited, highlighting the need for large-scale studies to confirm the favorable long-term outcomes after ESD.

## 5. Conclusions

In conclusion, early gastric LELC demonstrates favorable long-term outcomes after ESD despite frequently failing to meet current ESD curability criteria, primarily due to deep submucosal invasion. Considering these favorable long-term outcomes despite a low curative resection rate, early gastric LELC fulfilling the conventional curative criteria of current guidelines can be regarded as having been curatively treated by ESD, particularly in patients with high surgical morbidity. Further evidence is needed to determine whether patients who exceed the current curability criteria can be safely managed without additional surgery after ESD.

## Figures and Tables

**Table 1:** Comparison of the clinicopathological characteristics of early gastric LELC versus WD or MD EGC.

Characteristic	LELC	WD or MD EGC	*p*-Value
	(n = 53)	(n = 9014)	
Age (years)			0.092
Mean ± SD	62.47 ± 12.01	64.98 ± 9.64	
Median (range)	62 (37–86)	65 (27–98)	
Sex (%)			0.575
Male	41 (80.4)	6354 (77.1)	
Female	10 (19.6)	1889 (22.9)	
Location of tumor (%)			<0.001
Lower third (antrum, pylorus)	4 (7.5)	5220 (57.9)	
Middle third (angle, LB, MB)	21 (39.6)	2866 (31.8)	
Upper third (Fundus, cardia, EGJ, HB)	28 (52.9)	928 (10.3)	
Tumor size (pathology), cm			0.239
Mean ± SD	1.70 ± 0.89	1.61 ± 1.01	
Median (range)	1.4 (0.2–4.4)	1.4 (0.1–11.0)	
Tumor depth (%)			<0.001
Mucosa	10 (18.9)	7449 (82.6)	
SM1	2 (3.8)	708 (7.9)	
SM2 and SM3	41 (77.3)	856 (9.5)	
Lateral margin (%)			1.000
Negative	52 (98.1)	8623 (95.7)	
Positive	1 (1.9)	329 (3.6)	
Undetermined	0 (0)	62 (0.7)	
Vertical margin (%)			0.221
Negative	50 (94.3)	8748 (97.0)	
Positive	3 (5.7)	204 (2.3)	
Undetermined	0 (0)	62 (0.7)	
Lymphatic invasion (%)			0.376
Absent	50 (94.3)	8187 (90.8)	
Present	3 (5.7)	827 (9.2)	
Venous invasion (%)			0.491
Absent	53 (100)	8934 (99.1)	
Present	0 (0)	80 (0.9)	

LELC, lymphoepithelioma-like carcinoma; SD, standard deviation; ESD, endoscopic submucosal dissection; WD, well-differentiated; MD, moderately differentiated; EGC, early gastric cancer; LB, low body; MB, mid body; HB, high body; EGJ, esophagogastric junction.

**Table 2:** Detailed clinicopathological characteristics of patients with early gastric LELC.

Characteristic	LELC
	(n = 53)
Macroscopic type (%)	
I	1 (1.8)
IIa	3 (5.7)
IIb	3 (5.7)
IIc	18 (34.0)
III	0 (0)
Mixed type (IIa + IIc, etc.)	27 (51.0)
SMT-like	1 (1.8)
EBV status (%)	
Yes	44 (83.0)
No	6 (11.3)
Not done	3 (5.7)
Current *H. pylori* infection status (%)	
Yes	7 (13.2)
No	33 (62.3)
Not done	13 (24.5)
Pre-ESD biopsy (%)	
WD	2 (3.8)
MD	39 (73.6)
PD or PCC	12 (22.6)
Others	0 (0)

LELC, lymphoepithelioma-like carcinoma; SMT, submucosal tumor; EBV, Epstein–Barr virus; *H. pylori*, Helicobacter pylori; ESD, endoscopic submucosal dissection; WD, well-differentiated; MD, moderately differentiated; PD, poorly differentiated; PCC, poorly cohesive carcinoma.

**Table 3:** Short-term outcomes of ESD for early gastric LELC versus WD or MD EGC.

Characteristic	LELC	WD or MD EGC	*p*-Value
	(n = 53)	(n = 9014)	
En bloc resection			0.211
Yes	50 (94.3)	8746 (97.0)	
No	3 (5.7)	268 (3.0)	
R0 resection			0.578
Yes	49 (92.5)	8443 (93.7)	
No	4 (7.5)	71 (6.3)	
En bloc with R0 resection			0.450
Yes	47 (88.7)	8260 (91.6)	
No	6 (11.3)	754 (8.4)	
* Complete resection			0.704
Yes	44 (83.0)	7623 (84.6)	
No	9 (17.0)	1391 (15.4)	
** Curative resection (Diff-type)			<0.001
Yes	11 (20.8)	7292 (80.9)	
No	42 (79.2)	1722 (19.1)	
** Curative resection (Undiff-type)			<0.001
Yes	15 (28.3)	7292 (80.9)	
No	38 (71.7)	1722 (19.1)	
Bleeding			0.003
Absent	47 (88.7)	8767 (97.3)	
Present	6 (11.3)	247 (2.7)	
Perforation			1.000
Absent	53 (100)	8878 (98.5)	
Present	0 (0)	136 (1.5)	

LELC, lymphoepithelioma-like carcinoma; WD, well-differentiated; MD, moderately differentiated; EGC, early gastric cancer; Diff-type, differentiated-type; Undiff-type, undifferentiated-type. * Complete resection, en bloc resection with negative lateral and vertical resection margins, and without lymphovascular or perineural invasion. ** Curative resection, en bloc resection with negative lateral and vertical margins and no lymphovascular invasion, which fulfills eCura A, B by the Japanese gastric cancer treatment guidelines 2021, 6th edition [9].

## Data Availability

The data presented in this study are available upon request from the corresponding author. The data are not publicly available due to privacy and ethical restrictions.

## Supplementary Materials

The following supporting information can be downloaded at: https://www.mdpi.com/article/10.3390/cancers17183050/s1, Table S1: Clinical and Procedural Characteristics of 15 Patients with Risk Factors Managed with Observation After ESD.
