# Supplementary material for: Clinicopathological Features and Outcomes of Endoscopic Submucosal Dissection for Early Gastric Lymphoepithelioma-like Carcinoma
Source: Cancers (Basel). 2025 Sep 18;17(18):3050. doi: 10.3390/cancers17183050 (PMC12469100; doi:10.3390/cancers17183050)
Supplement: Supplementary file 1 [file cancers-17-03050-s001.zip › cancers-3855479-supplementary.pdf]

Table S1. Clinical and Procedural Characteristics of 15 Patients with Risk Factors Managed with Observation After ESD.

No.	Age	Sex	Location	Size (cm)	SM invasion (μm)	Lateral margin involvement	Vertical margin involvement	Lym invasion	Vas invasion	Further management	Death
1	53	Female	HB	1	700	Negative	Negative	Negative	Negative	Close surveillance	No
2	54	Male	HB	3	1500	Negative	Negative	Negative	Negative	Close surveillance	No
3	56	Female	Cardia	1	250	Negative	Negative	Negative	Negative	F/U as scheduled	No
4	59	Male	LB	1.8	129	Negative	Negative	Negative	Negative	F/U as scheduled	No
5	62	Male	HB	3.2	2000	Negative	Negative	Negative	Negative	Refused surgery (Comorbidities; AAA, IHD)	No
6	65	Male	MB	0.6	1800	Negative	Negative	Negative	Negative	Close surveillance	No
7	67	Male	MB	0.6	500	Negative	Negative	Negative	Negative	Refused surgery (Comorbidities; IHD)	No
8	69	Male	HB	2.4	700	Negative	Negative	Negative	Negative	Refused surgery (Advanced age)	No
9	69	Male	Cardia	1	557	Negative	Negative	Negative	Negative	Close surveillance	No
10	72	Male	LB	1.4	500	Negative	Negative	Negative	Negative	Refused surgery (Advanced age)	No
11	73	Male	MB	0.8	900	Negative	Negative	Negative	Negative	Refused surgery (Advanced age)	No
12	76	Male	Angle	1.4	1800	Negative	Negative	Positive	Negative	Refused surgery (Advanced age)	No
13	80	Male	HB	2.8	650	Negative	Negative	Negative	Negative	Refused surgery (Advanced age)	No
14	82	Male	Cardia	1.2	4000	Negative	Negative	Negative	Negative	Refused surgery (Advanced age)	Yes
15	85	Male	Cardia	2.2	2300	Negative	Positive	Negative	Negative	Refused surgery (Comorbidities; COPD, Lymphoma)	Yes

SM, submucosal; Lym, lymphatic; Vas, vascular; HB, high body; low body; MB, mid body; F/U, follow-up; AAA, abdominal aortic aneurysm; IHD, ischemic heart disease; COPD, chronic obstructive pulmonary disease.

Note: Risk factors for residual tumor or lymph node metastasis were defined as the presence of one or more of the following: pT1b, positive resection margin, or lymphovascular invasion.
